# Assessing Histology Structures by *Ex Vivo* MR Microscopy and Exploring the Link Between MRM-Derived Radiomic Features and Histopathology in Ovarian Cancer

**DOI:** 10.3389/fonc.2021.771848

**Published:** 2022-01-19

**Authors:** Marion Tardieu, Yulia Lakhman, Lakhdar Khellaf, Maida Cardoso, Olivia Sgarbura, Pierre-Emmanuel Colombo, Mireia Crispin-Ortuzar, Evis Sala, Christophe Goze-Bac, Stephanie Nougaret

**Affiliations:** ^1^ Montpellier Cancer Research Institute (IRCM), INSERM U1194, University of Montpellier, Montpellier, France; ^2^ Department of Radiology, Memorial Sloan Kettering Cancer Center, New York, NY, United States; ^3^ Department of Pathology, Montpellier Cancer Institute (ICM), Montpellier, France; ^4^ BNIF Facility, L2C, UMR 5221, CNRS, University of Montpellier, Montpellier, France; ^5^ Department of Surgery, Montpellier Cancer Institute (ICM), Montpellier, France; ^6^ Cancer Research UK, Cambridge Institute, University of Cambridge, Cambridge, United Kingdom; ^7^ Department of Radiology, University of Cambridge, Cambridge, United Kingdom; ^8^ Department of Radiology, Montpellier Cancer Institute (ICM), Montpellier, France

**Keywords:** ovarian cancer, MRI, radiomics, machine learning, histology

## Abstract

The value of MR radiomic features at a microscopic scale has not been explored in ovarian cancer. The objective of this study was to probe the associations of MR microscopy (MRM) images and MRM-derived radiomic maps with histopathology in high-grade serous ovarian cancer (HGSOC). Nine peritoneal implants from 9 patients with HGSOC were imaged *ex vivo* with MRM using a 9.4-T MR scanner. All MRM images and computed pixel-wise radiomics maps were correlated with the slice-matched stroma and tumor proportion maps derived from whole histopathologic slide images (WHSI) of corresponding peritoneal implants. Automated MRM-derived segmentation maps of tumor and stroma were constructed using holdout test data and validated against the histopathologic gold standard. Excellent correlation between MRM images and WHSI was observed (Dice index = 0.77). Entropy, correlation, difference entropy, and sum entropy radiomic features were positively associated with high stromal proportion (r = 0.97,0.88, 0.81, and 0.96 respectively, *p* < 0.05). MR signal intensity, energy, homogeneity, auto correlation, difference variance, and sum average were negatively associated with low stromal proportion (r = –0.91, –0.93, –0.94, –0.9, –0.89, –0.89, respectively, *p* < 0.05). Using the automated model, MRM predicted stromal proportion with an accuracy ranging from 61.4% to 71.9%. In this hypothesis-generating study, we showed that it is feasible to resolve histologic structures in HGSOC using *ex vivo* MRM at 9.4 T and radiomics.

## 1 Introduction

High-grade serous ovarian cancer (HGSOC) is the most prevalent histological subtype of ovarian cancer (1). Advanced-stage HGSOC is often approached with neoadjuvant chemotherapy to reduce tumor burden followed by cytoreductive surgery with or without hyperthermic intraperitoneal chemotherapy (HIPEC) (2, 3). Response to neoadjuvant treatment at histopathology manifests as an increase in stromal tissue and decrease in tumor cells, but those changes cannot be assessed on standard cross-sectional imaging (4). Indeed, CT only provides anatomic information, whereas MRI captures both anatomic and functional data. Preliminary results in ovarian cancer suggest that the quantitative parameters derived from diffusion-weighted MR imaging (DWI-MRI) may serve as a biomarkers of cell density (5–7). For example, the increase in ADC values in HGSOC peritoneal implants (decrease in cell density) during neoadjuvant chemotherapy was associated with good treatment response as assessed by RECIST 1.1 criteria and CA125 level (8).

To date, few studies have explored the associations between CT/MR images which interrogate tumor at macroscopic scale and histopathologic images which depict tumor at microscopic scale. In contrast, studies have recently focused on the role of radiomics. Radiomic analysis extracts a large amount of quantitative data and, thus, has the potential to uncover salient features that are imperceptible to human observers yet possibly reflective of microscopic changes in tumor in response to treatment (9–13). Preliminary work in CT found that higher image-based tumor heterogeneity was associated with worse prognosis and greater risk of incomplete surgical resection in HGSOC (14).

However, image-based tumor heterogeneity and radiomics features have not yet been exactly correlated with findings at histopathology. Exploring the associations between image-based tumor heterogeneity and biologic underpinnings at histopathology would offer an important avenue for monitoring response in time and space non-invasively using imaging, as “virtual biopsy”. For example, it has been recently reported that a high content of stroma present in HGSOC was associated with a high pathologic stage at diagnosis and displayed a reduced overall survival and poor prognosis independently from the histology type (15, 16). For a long time, the potential role in carcinogenesis of stromal cells has been neglected, as they were regarded just as part of an inflammatory reaction induced by necrotic cancer cells. It is now recognized that the stroma composition and architecture, in terms of vascularization, type of cells, and their secretion, play a role in the establishment and progression of cancer cells. It is also now well established that the stroma contributes to ovarian tumorigenesis and progression (17). Concerning the recent clinical radiomics model, Lu et al. found a radiomics model associated with DFS and genomic pathway (18). This radiomic model was positively correlated with a stroma marker, the fibronectin, and associated with a proportion of tumor-associated stromal cells and patient prognosis (18). Being able to assess the stroma–tumor ratio in a non-invasive way with radiomics may open a new pathway in assessing tumor response in HGSOC.

Beyond the analysis of standard and functional MR imaging, *ex vivo* high-field MRI (i.e., magnetic resonance microscopy, MRM) offers a unique opportunity to probe the tumor at the microscopic scale because MRM can reach a resolution of 40 µm. Although much lower than that of optical microscopy (0.25 µm), this resolution is significantly higher compared to standard MRI (1 mm) allowing the visualization of histological details. Work performed *ex vivo* at 7.0 T was able to visualize distinctive features of both benign and malignant lesions in breast tissue (19). In prostate, Fan et al. have evaluated the feasibility of 9.4-T *ex vivo* MRI to guide pathologists’ examination in the evaluation of prostate cancer (20). They demonstrated excellent anatomical detail as well as significant T2 values and ADC differences between cancer and normal prostatic tissues (20). Durand et al. performed *ex vivo* MRI with direct histological correlation of the prostate gland that approached histological spatial resolution enabling the visualization of gland microanatomy with MRM (21). More recently, a study has investigated the ability of *ex vivo* 7.0-T MRI to localize prostate cancer and to predict the margin status in fresh radical prostatectomy specimens using histology as the reference standard (22). The author found that *ex vivo* MRI was able to accurately localize prostate cancer in radical prostatectomy specimens, and the technique provided information on the margin status (22). In brain tumors, Martinez-Bisbal et al. combined MRM with MR spectroscopy to study metabolites of *ex vivo* tumors at high resolution (23). However, to our knowledge the role of MRM in ovarian cancer remains unexplored.

The aim of this proof-of-concept study was to evaluate the correlation between tumor-stroma maps derived from MRM and whole histopathology slide images (WHSI) of histopathologic specimens and to develop an automated visual map of stromal proportion in HGSOC peritoneal implants using quantitative analysis of MRM images.

## 2 Materials and Methods

### 2.1 Patient Inclusion

**Figure 1:**
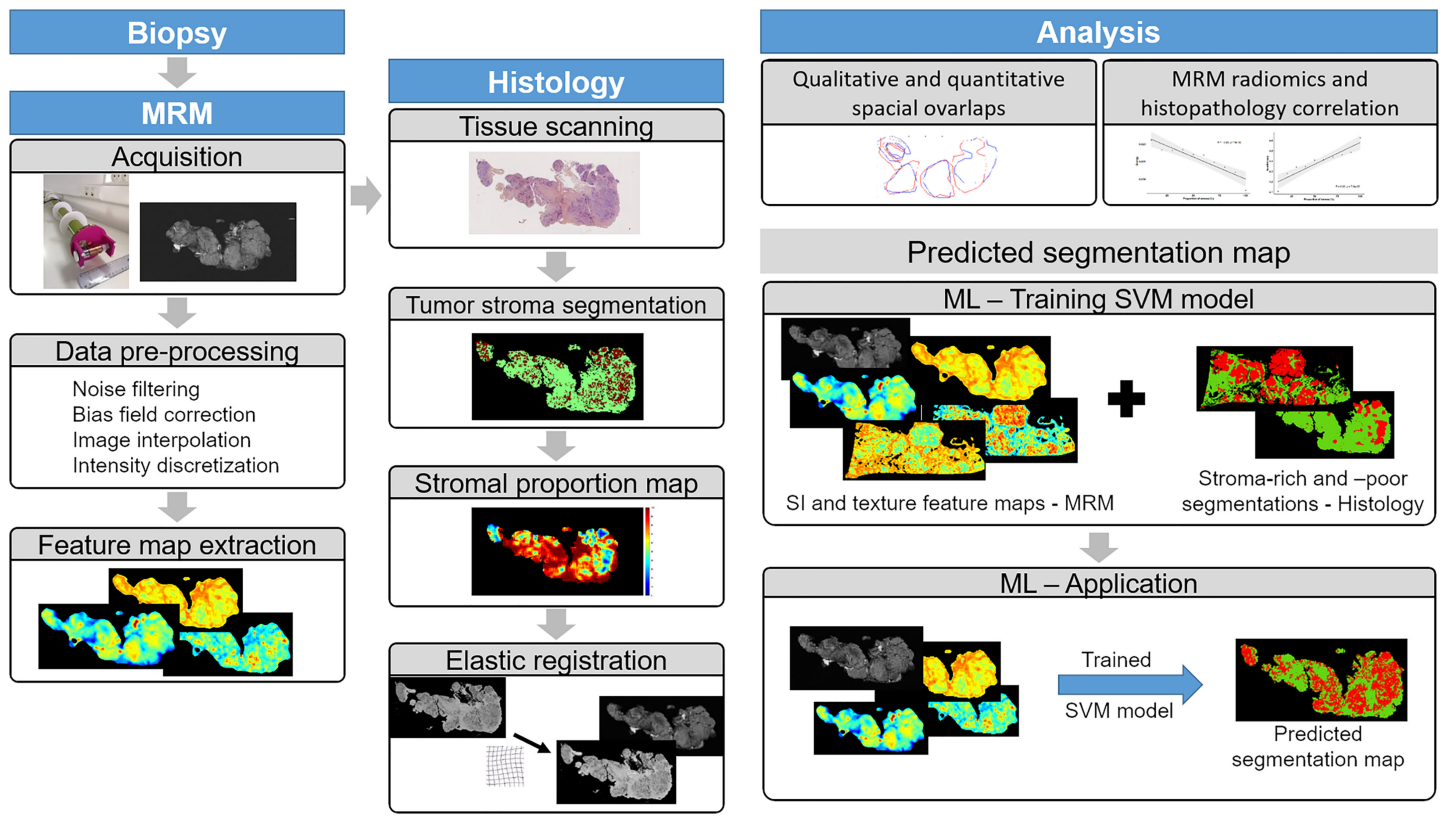
Illustration of the study experimental workflow.

Nine patients (mean age 65.6 ± 8.2 years) with advanced HGSOC referred to surgery were included in the study; among them, 2 had primary debulking surgery and 7 had interval debulking surgery after neoadjuvant chemotherapy. Patient characteristics are summarized in **Table 1**. The Institutional Review Board approved this prospective study, and all patients signed the written informed consent form prior to enrollment. **Figure 1** shows the study experimental workflow.

**Table 1:** Patients characteristics.

**Patient age (mean + SD)**	65.6 ± 8.2
**BRCA-mutant**	
**Yes**	1
**No**	8
**Neoadjuvant chemotherapy**	
**Yes**	7
**No**	2

### 2.2 Biopsy Preparation and Evaluation

For each patient, index peritoneal implant was selected *a priori* based on the review of preoperative MRI in conjunction with the surgeon. Resected implants were transported from the operating room to the pathology laboratory per routine procedure where they were transformed into 3 × 1.5 × 1 cm^3^ blocks and fixed in formalin solution. Prior to MRM acquisitions, fixed specimens were soaked in saline solution with 1% Gd-BOPTA (MultiHance; Bracco Imaging, Milan, Italy) during 1 h. The implants were then laid on a plastic plate inside a tube filled with a perfluorocarbon solution (Fluorinert FC-40, 3M™ Electronic Liquids, Saint Paul, USA) to reduce susceptibility artifacts induced by air. After imaging, the specimens were transported back to the pathology laboratory for histological evaluation.

### 2.3 MRM Technique and Radiomic Feature Extraction

MRM acquisitions were performed on a 9.4-T MR scanner (Agilent Varian 9.4/160/ASR, CA, USA) associated with the VnmrJ imaging acquisition system (Agilent, Palo Alto, California, USA) and using a dedicated ribbon solenoid coil (24). MRI experiments included two sets of acquisitions: a high-resolution and a 90 × 90 × 180 µm^3^ T1-weighted fat-suppressed gradient echo images. The later set of images was selected to facilitate radiomic analysis by allowing to interpolate images to an isotropic voxel spacing with a minimum interpolation factor (25, 26). All MRM image acquisition parameters are summarized in **Table 2**. Prior to radiomic analysis, MRM images were preprocessed using the open-source software 3D slicer (27) (http://www.slicer.org) and by following IBSI guidelines (26). Briefly, noise filtering, bias field correction, image interpolation to isotropic voxel size, and intensity outlier filtering [*µ* ± 3*σ*] were performed. Texture feature maps were then extracted on a per-pixel basis with an in-house software implemented in Matlab (The MathWorks, Natick, MA, USA). The gray-level co-occurrence matrix (GLCM) (28) was computed in 2D for each pixel using the 3 neighboring pixels of each direction around it. The dynamic range of signal intensities was reduced to 64 gray levels. The GLCM, *p(i,j)*, represents the spatial relationship of pixels by measuring the occurrence between a pixel ***i*** with a certain intensity with a pixel ***j*** of another intensity along the 13 directions in 3D. Thirteen Haralick feature maps were extracted from GLCM: energy, contrast, entropy, homogeneity, dissimilarity, correlation, variance, sum average, sum entropy, difference variance, difference entropy, autocorrelation, and cluster tendency. The total time acquisition was 1 h, and images were obtained usually within 3 h of resection.

**Table 2:** Sequences parameters.

Acquisition name	High-resolution	For radiomics analysis
Tr/TE (ms)	2000/9.14	
Flip angle	60°	
Averages	4	
Matrix	512 × 256	
FOV	From 26 × 13 to 32 × 16 mm^2^	46 × 23 mm^2^
Resolution	From 51 × 51 to 62.5 × 62.5 µm^2^	90 × 90 µm^2^
Slice/thickness	30/300 µm	40/180 µm
Scan time	34 min 12 s	17 min 8 s

#### 2.3.1 Tissue Scanning

After MRM experiments, each block was sent to the pathology laboratory and cut into 4-µm sections parallel to MR sections, discerned by the plastic plate. Histologic specimens were stained using hematoxylin–eosin–saffron (HES) stain, and whole-slice sections were then scanned into digital data with an automated whole-slide scanner (NanoZoomer-XR scanner C12000, Hamamatsu, Japan), at ×20 magnification and a pixel size of 0.46 µm.

#### 2.3.2 Tumor Stroma Segmentation

Stoma and tumor regions on WHSI were identified and segmented with the open-source software QuPath (29). In order to correlate MRM and WHSI, as MR texture features were estimated for each pixel, stromal proportion was also locally calculated. Thus, from the tumor-stroma segmentation WHSI map, stromal proportion was assigned to each pixel by measuring this proportion in a circular neighborhood with a radius of 3 pixels. Stromal proportion calculation was not possible for two of the nine specimens. One of them had mostly fat tissue, causing too few tumor and stroma tissues to be correlated with corresponding MRM slices (specimen iii. on **Supplementary Material Figure S1**). The second tumor was resected after chemotherapy and had a significant necrosis, with the consequence that neither tumor tissue nor stroma remained (specimen iv. on **Supplementary Material Figure S1**).

#### 2.3.3 Elastic Registration

**Figure 2:**
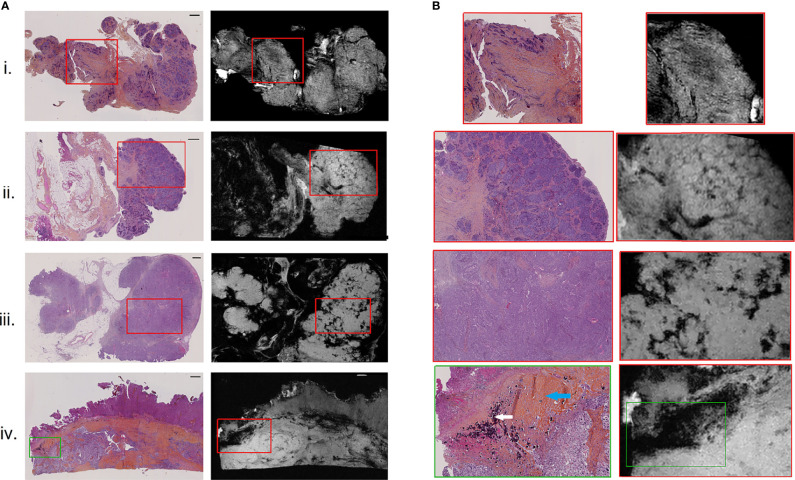
H&E-stained histological images **(A)**, left) with corresponding high-resolution MR images **(A)**, right), for 4 of the 9 resected peritoneal implants (i–iv). Magnified regions **(B)** of histological (left) and high-resolution MR (right) images, indicating by red and green boxes on **(A)**. Scale bar at top right of histological images **(A)** is 1 mm. White and blue arrows on the histologically magnified region **(B)**. (iv) Indicated respectively psammoma bodies and hyaline stroma.

Due to histological fixation and sectioning, elastic deformations existed between histology sections and corresponding MRM slices. To remedy this and compare those images at the corresponding pixel, manual 3D non-linear co-registration was performed using the 3D slicer. Three specimens could not be precisely co-registered: deformations were too important, making the precise pixel-wise registration impossible between WHSI and MRM images (specimen ii. on **Figure 2** and specimens ii. and v. on **Supplementary Material Figure S1**).

### 2.4 Statistical Analysis

#### 2.4.1 MRM Image and WHSI Comparison

A qualitative visual assessment of high-resolution MR images and texture maps with their corresponding WHSI of the nine *ex vivo* peritoneal implants was evaluated by the pathologist and the radiologist. Spatial overlaps between WHSI and MRM images were measured and quantified using the Dice similarity coefficient using the following formula: *DSC* = 2 (*A* ∩ *B*) / (*A* + *B*).

#### 2.4.2 MRM Radiomics and Histopathology Pixel-Wise Correlation

For the MRM and histology correlation, 5 implants had to be excluded: three of them could not be precisely co-registered, two for which the stromal proportion maps could not be extracted. Clinical characteristics of these four remaining tumors were 0 BRCA mutant and 2 obtained after neoadjuvant chemotherapy. MRM signal intensity (SI) and texture maps were compared pixel by pixel to the stromal proportion map from WHSI. Stromal proportion maps from WHSI were divided into increments of 10 equal percentage points, and mean SI and texture values were computed for these 10 regions. Pearson coefficients were calculated to evaluate the correlation between mean texture feature values and stromal proportion. *p*-values less than 0.05 were considered statistically significant. All statistical analyses were performed with R software version 4.0.0.

#### 2.4.3 Predicted MR Segmentation Maps

Pixels of the four remaining tumors were classified as stroma-rich, i.e., stromal proportion >50%, and stroma-poor, i.e., stromal proportion <50%, Of these pixels, 30% were randomly selected from the four tumors, to generate a classification table of 58,945 pixels associated with 14 inputs (13 texture features and the label). Of these selected pixels, 50% were randomly chosen to train the machine learning model with balanced number of stroma-rich and -poor pixels; the 50% remaining pixels were used to test the algorithm (29,473 pixels). The classification model was completed using the support vector machine (SVM) classifier and 20-fold cross-validation, with the classification learner toolbox of Matlab 2020a. SVM is a supervised learning model that classifies by mapping the input data into a higher-dimensional space allowing to find a hyperplane separator. The SVM training model was exported and used to measure the confusion matrix and accuracy value on the test set. Finally, this trained algorithm was applied to the 4 tumors separately to generate predicted segmentation maps and were compared to the stroma-rich and -poor segmentations, extracted from the histopathologic images.

## 3 Results

### 3.1 Comparison Between MRM and Histopathology Reading

#### 3.1.1 Visual Assessment, Qualitative Analysis

All nine implants were evaluated for visual MRM assessment and subsequently compared with histopathology. In all 9 cases, the pathologist was able to identify different relevant histological structures (i.e., tumor, stroma and fat) on MRM images. On MRM images, tumor cells appeared as high signal intensity areas separated by lower signal intensity foci representing fibrous stroma (**Figure 2**.i). In some cases, massive tumor infiltration appeared clustered in lobules separated by stroma, giving the appearance of a cauliflower (**Figure 2**.ii), well recognized on both MRM and histology images. Interestingly, three histological structures resulted on signal loss on MRM images. Glandular lumens, forming slit-like spaces inside dense tumor regions (**Figure 2**.iii), and psammoma bodies that are round collections of calcium did not produce any MR signal (**Figure 2**.iv, white arrow). Finally, regions of hyaline stroma, composed of hypocellular old collagen, were also associated with a signal loss area in MRM images (**Figure 2**.iv, blue arrow). Histology and MRM images of the implants not shown in **Figure 2** are presented on **Supplementary Material Figure S1**.

#### 3.1.2 Quantitative Analysis

**Figure 3:**
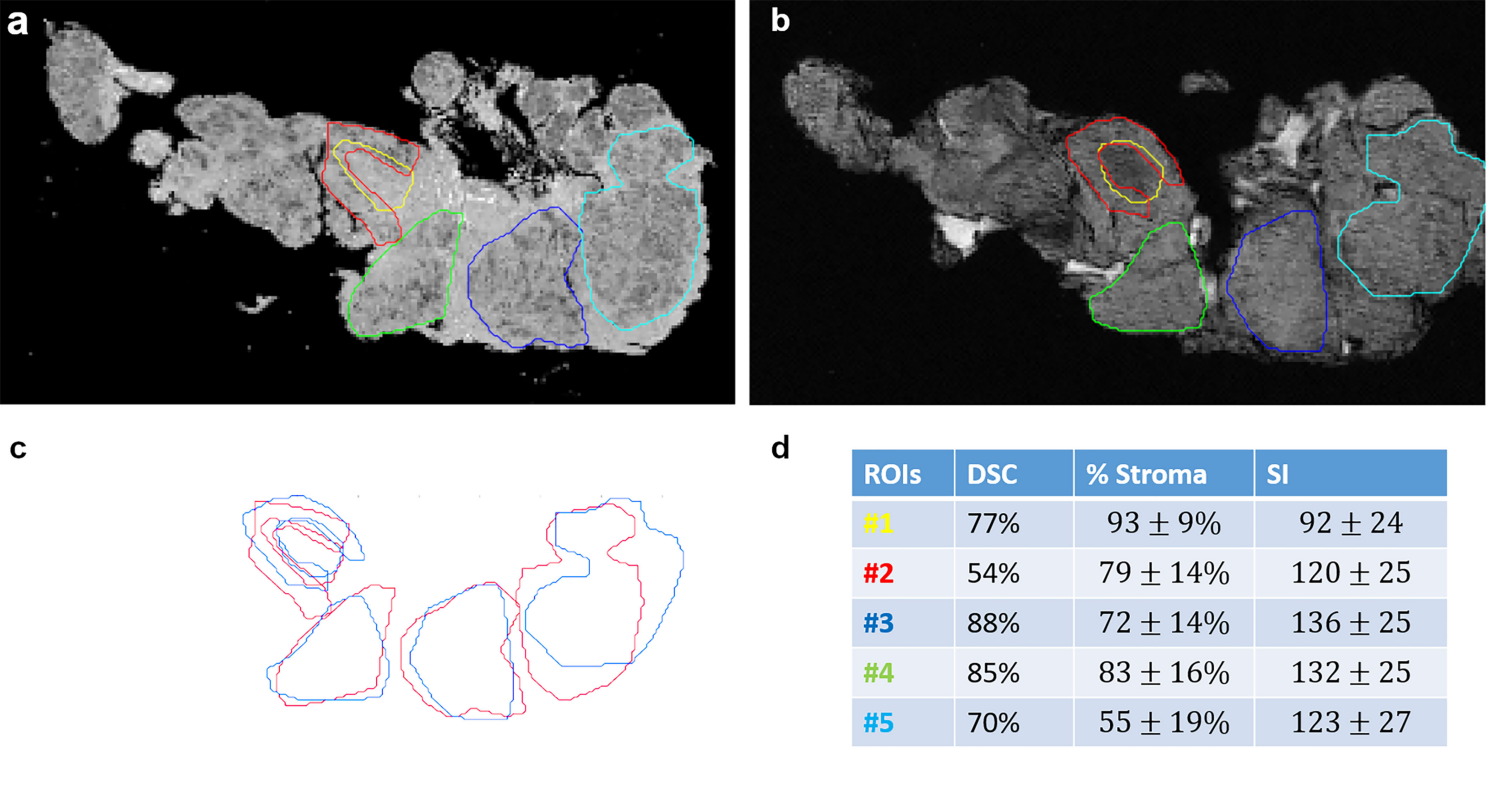
Spatial overlaps between histological images **(A)** and MR images **(B)** for stroma and tumor portion. ROIs were manually drawn on MRM and whole slide images and then superposed in **(C)**. Dice similarity index (DSI), stroma proportion, and signal intensity are presented in a table **(D)** for each ROI.

A spatial overlap between tumor, stroma, and fat regions on MRM images and at histopathology was measured (**Figure 3**). The Dice similarity coefficient was 77% for the entire dataset.

### 3.2 Association Between Radiomics and Histopathology Features: Quantitative Assessment

**Figure 4:**
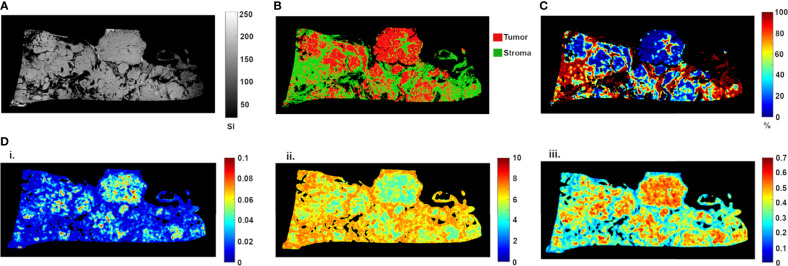
High-resolution MR image **(A)** of resected peritoneal implant with corresponding tissue segmentation map **(B)** and stromal proportion map **(C)**, in %). Corresponding texture maps **(D)** for energy (i), entropy (ii), and homogeneity (iii) features. For this implant, high tumor proportion (low proportion of stroma) was associated with higher energy (from 0.029 ± 0.020 to 0.019 ± 0.014, t-test p = 0.0002), homogeneity (from 0.44 ± 0.10 to 0.38 ± 0.10, t-test p = 0.0001) and signal intensity (from 141.41 ± 28.30 to 116.09 ± 41.52, t-test p = 0.0002) scores (**Figure 3**). In contrast, high stromal proportion (low tumor proportion) was associated with higher entropy score (from 5.87 ± 1.00 to 6.47 ± 0.83, t-test p = 0.0002).

**Figure 5:**
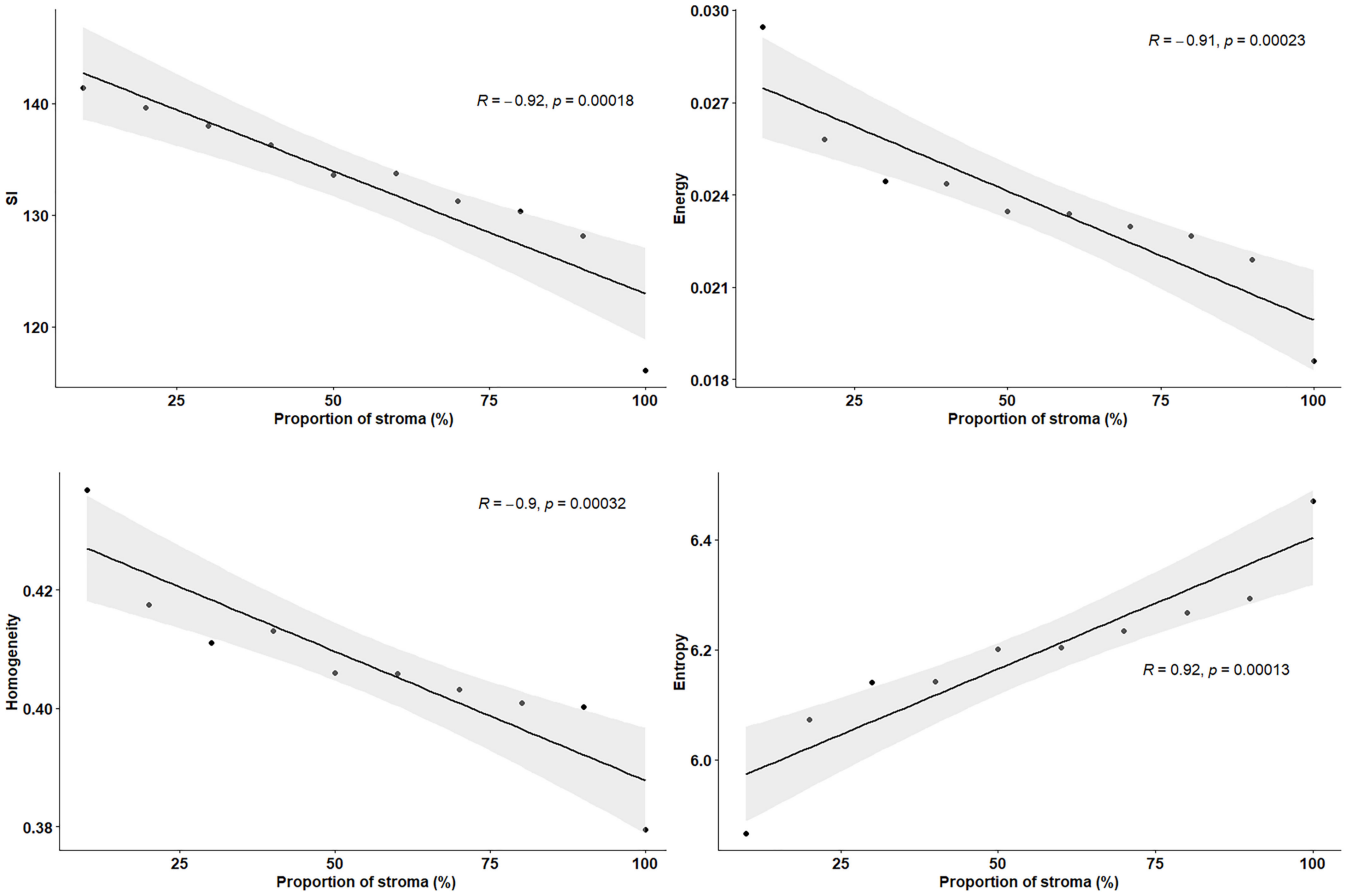
Pearson’s correlation plots between texture features and proportion of stroma (%), extracted from resected peritoneal implant from **Figure 2**. Stromal proportion map was divided into increments of 10 percentage points and mean texture values were calculated for these 10 regions. r indicates the correlation coefficient.

*Ex vivo* MRM radiomics features were extracted, and texture maps were generated for each of the four implants. **Figure 4** illustrates energy, entropy, and homogeneity maps from a representative peritoneal carcinomatosis implant, with corresponding tumor-stroma segmentation and stromal proportion maps. The stromal proportion map was divided into increments of 10 percentage points, and mean texture values were calculated for these 10 regions. Correlation plots between mean texture feature values and stromal proportion were constructed (**Figure 5**), and Pearson’s correlation coefficients with corresponding p-values were then determined (**Table 3**). Pearson correlation coefficients ranged from 0.47 to 0.97 with p-values significant for 10 of 13 features. Entropy, correlation, difference entropy, and sum entropy radiomic features were positively associated with high stromal proportion (r = 0.97, 0.88, 0.81, and 0.96, respectively, *p* < 0.05). MR signal intensity, energy, homogeneity, auto correlation, difference variance, and sum average radiomic features were negatively associated with low stromal proportion and as such linked with higher tumor proportion (r = –0.91, –0.93, –0.94, –0.9, –0.89, and –0.89, respectively, *p* < 0.05).

**Table 3:** Pearson’s correlation (r) between texture features and stromal proportion.

	SI	Constrat	Dissimilarity	Energy	Entropy
r	-0.91	-0.76	0.47	-0.93	0.97
*p*-value	<0.0001	0.011	0.17	<0.0001	<0.0001
	**Homogeneity**	**AutoCorrel**	**ClusterTend**	**Correlation**	**DiffEntropy**
r	-0.94	-0.9	0.68	0.88	0.81
*p*-value	<0.0001	0.0005	0.029	0.0007	0.0046
	**DiffVariance**	**SumAver**	**SumEntropy**	**Variance**	
r	-0.89	-0.89	0.96	0.56	
*p*-value	0.0005	0.0006	<0.0001	0.093	

### 3.3 Estimated Segmentation Map

**Figure 6:**
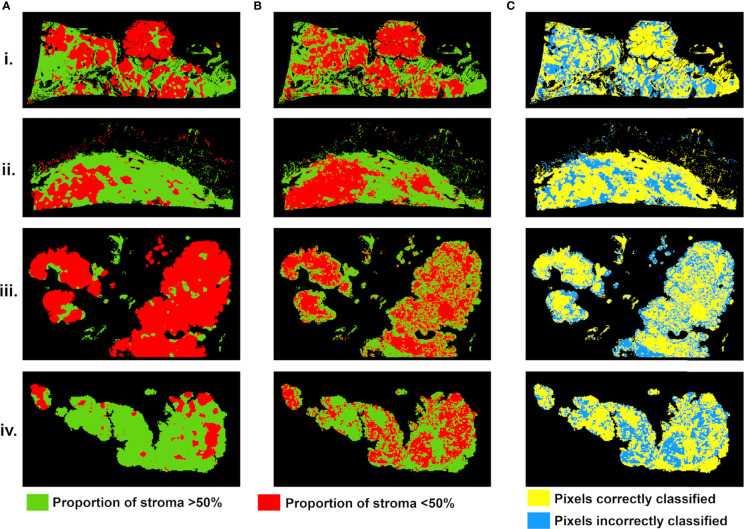
Stroma-rich (>50%) and -poor (<50%) histological segmentations **(A)** and predicted segmentation map from MR feature maps **(B)**, with pixels in green where stromal proportion >50% (stroma-rich) and in red where stromal proportion <50% (stroma-poor). **(C)** Evaluation map with pixels correctly classified in yellow and incorrectly classified in blue. For four tumors from (i–iv).

Stroma-rich and -poor histological segmentations (proportion of stroma >50% and <50%) were defined from the stromal proportion map, allowing to train an SVM model with MR texture maps as inputs. Boxplots showing classified features values between >50% and <50% are presented in supplementary material **Figure S2**. The estimated segmentation maps were performed for the 4 samples, using the training SVM model, and are presented in **Figure 6** with their corresponding stroma-rich and -poor histological segmentations, allowing confusion matrix calculation (**Table 4**). An accuracy for predicting stromal proportion from MRM images ranged from 61.4% to 71.9% on the holdout test data.

**Table 4:** Confusion matrix results with TPR, true positive rate; TNR, true negative rate; FPR, false positive rate; FNR, false negative rate.

	Accuracy	TPR	TNR	FPR	FNR
Test set	66.6%	70.6%	62.4%	37.6%	29.4%
i.	63.3%	65.9%	60.5%	39.5%	34.1%
ii.	71.9%	82.5%	66.4%	33.6%	17.5%
iii.	62.1%	60.9%	73.4%	26.6%	39.1%
iv.	61.4%	71.3%	59.4%	40.7%	28.7%

## 4 Discussion

This study demonstrates the feasibility of obtaining *ex vivo* images of peritoneal implants from HGSOC at 9.4 T at a resolution of 40 µm. Slice-matched MRM demonstrated strong structural similarities compared to whole-slide histology specimens. Tumor, stroma, and adipose tissues were all apparent on MRM images, confirmed on their slice-matched histological samples. In addition, we found that MRM-derived radiomic features were able to discriminate tumor tissue, stroma, and adipose structures and could evaluate the stroma–tumor ratio.

To date, few studies have imaged tumor at a microscopic scale. Preliminary studies in breast, prostate and brain cancer demonstrated excellent correlation between MRM images and histopathologic characteristics of malignant tissue (19–23, 30, 31). Similarly, for the first time in HGSOC, we were able to obtain a direct correlation between histology and MRM images at 9.4 T.

In our study, we also correlated radiomics features with the tumor/stromal proportion pixel by pixel, from four tumors. Besides the extensive work performed in MRI on radiomics in all fields of study, the link between data extracted from the image and the pathology results is still lacking as radiomics has never been evaluated at a microscopic scale. For example, Vargas et al. found with CT that inter- and intratumor heterogeneity was linked to poor prognosis in HGSOC (14). Rizzo et al. evaluated whether CT radiomics features extracted from the primary tumor alone or combined with clinical data were associated with residual tumor at surgery in 101 patients with ovarian cancer. They were able to predict the risk of disease progression within 12 months (11). The authors found that along with other features, homogeneity was associated with residual tumor at surgery. In our study, entropy, correlation, difference entropy, and sum entropy radiomics features were positively associated with stromal proportion while MR signal intensity, energy, homogeneity, auto correlation, difference variance, and sum radiomics features were positively associated with tumor proportion. The energy feature is high when GLCM contains only a few high-intensity pixels, which means that the MR image grayscale is uniform. In our study, the energy feature is high when the tumor region is mostly composed of high density of tumor cells. In contrast, entropy reflects the complexity of the MR image and its value is high when the MR image is disorderly. In this work, high entropy values matched with areas encircling tumor cells, mostly composed of stroma. Thus, we found that areas of tumor cells appeared uniform on MR images, while areas of stroma appeared heterogeneous. Those radiomics features associated with stroma heterogeneity may be explained by stroma neoangiogenesis known to be disorderly and the consistence of the stroma itself. Indeed, it mostly consists of heterogeneous cell types and a mixture of amorphous components. Various cell types are found in the stroma of HGSOC, including immune and inflammatory cells, endothelial cells, adipocytes, and the “cancer-associated fibroblasts”.

In our study, we built a model allowing to compute a visualize map of estimated stromal and tumor regions from MR images. This model was trained on 29,473 pixels with SI and 13 texture feature values as inputs and binary stromal proportion, extracted from histopathologic data, as targets. The 4 generated predicted segmentation maps were compared to the actual segmentation maps measured from histology and found a good accuracy (61.4 to 71.9). Relative low accuracy values can be explained by the presence of histological structures not taken into account in the model training, such as glandular lumens resulting in signal loss (**Figures 2**.iii, **Figure 6**.iii). These values can also be explained by the difference of slice thickness between MR and histological images, with a factor of 45%. Future studies will validate the algorithm with new samples prior to and after chemotherapy to assess tumor response and will try to translate it to clinical MRI.

Finally, predicted segmentation maps allowed us to assess the stroma–tumor ratio by *ex vivo* imaging. Multiple studies have demonstrated the importance of stroma in ovarian tumorigenesis, progression, and reduced overall survival (15–17). Our interesting results linking radiomics stroma heterogeneity and pathology may explain some of the radiomics associations found by Lu et al., where their radiomics model was correlated with fibronectin (18). Future ongoing work will try to translate this *ex vivo* findings to *in vivo* real-time evaluation from 1.5- or 3-T scanners used in clinical routine.

Our study has several limitations that can be attributed to its exploratory nature in which the feasibility of a new method was evaluated. The total sample size was rather small, limiting the statistical power. Moreover, from the 9 studied specimens, only four were exploited for the MRM and histology pixel-wise correlation, reducing this sample size. From the five excluded samples, three were removed because of the impossibility to precisely co-register WHSI and MRM images. One of the main elements that could explain this impossibility concerns fatty tissue. Indeed, intracellular fat droplets get dissolved during the preparation of HES slides. While this fatty tissue participates in maintaining the shape of the tumor, its dissolution can lead to important deformation of the shape in 3D. However, performing a pixel-wise correlation allowed us to compensate this weakness by increasing the number of information.

Only one pathologist and radiologist reviewed the image. Regarding radiomics analysis, we limited the number of features extracted to relatively basic texture features giving the small number of samples. Finally, we only evaluate the stroma tumor globally. As the stromal component contains a mixture of cells of different origins, the exact elements in the stroma measured by radiomics remain unclear. A study to associate radiomics features with each component in stroma including fibroblast activation, immune cell infiltration, and extracellular matrix density is necessary to better understand the link between radiomics and stromal proportion.

## 5 Conclusion

In conclusion, MRM can be optimized to achieve high-resolution images of HGSOC with images obtained within 3 h of resection. This technique offers the possibility of providing valuable information to surgeons in the intraoperative setting. Furthermore, MRM with radiomics analysis allowed us to associate radiomics features to tumor and stromal proportion.

## Data Availability Statement

The raw data supporting the conclusions of this article will be made available by the authors, without undue reservation.

## Ethics Statement

The studies involving human participants were reviewed and approved by the local ethics committee. The patients/participants provided their written informed consent to participate in this study.

## Author Contributions

Conceptualization, MT, YL, MC, CG-B, and SN. Data curation, MT, LK, CG-B, and SN. Formal analysis, MT, LK, MC, P-EC, CG-B, and SN. Funding acquisition, MT and SN. Investigation, MT, YL, MC, OS, and SN. Methodology, MT, MC, P-EC, MC-O, C-GB, and SN. Project administration, MT and SN. Resources, MT, LK, OS, P-EC, CG-B, and SN. Software, MT, LK, MC, P-EC, and SN. Supervision, MT, MC-O, ES, and SN. Validation, MT, MC-O, ES, and SN. Visualization, MT, MC-O, ES, and SN. Writing—original draft, MT, YL, ES, and SN. Writing—review and editing, MT, YL, LK, OS, P-EC, MC-O, ES, and SN. All authors contributed to the article and approved the submitted version.

## Funding

This research was funded by Foundation de l’Avenir grant and SIRIC Montpellier Cancer Grant INCa-DGOS-Inserm_12553.

## Conflict of Interest

The authors declare that the research was conducted in the absence of any commercial or financial relationships that could be construed as a potential conflict of interest.

## Publisher’s Note

All claims expressed in this article are solely those of the authors and do not necessarily represent those of their affiliated organizations, or those of the publisher, the editors and the reviewers. Any product that may be evaluated in this article, or claim that may be made by its manufacturer, is not guaranteed or endorsed by the publisher.

## Supplementary Material

Supplementary Figure 1 H&E stained histological images (left) with corresponding high-resolution MR images (right), for 5 of the 9 resected peritoneal implants (i. to v.).

Supplementary Figure 2 Box plots showing feature values between pixels labeled as stromal proportion greater than 50% and as less than 50%, measured from the four available HGSOC peritoneal implants (total of 196073 classified pixels). Wilcoxon test, *p*-values <0.0001, for the 4 presented features.

## References

[1] Torre LA Trabert B DeSantis CE Miller KD Samimi G Runowicz CD. Ovarian Cancer Statistics, 2018. CA: A Cancer J Clin (2018) 68(4):284−96. doi: 10.3322/caac.21456 PMC6621554 29809280

[2] Lee J-M Minasian L Kohn EC. New Strategies in Ovarian Cancer Treatment. Cancer (2019) 125(S24):4623−9. doi: 10.1002/cncr.32544 31967682 PMC7437367

[3] van Driel WJ Koole SN Sikorska K Schagen van Leeuwen JH Schreuder HWR Hermans RHM. Hyperthermic Intraperitoneal Chemotherapy in Ovarian Cancer. N Engl J Med (2018) 378(3):230−40. doi: 10.1056/NEJMoa1708618 29342393

[4] Jacob F Marchetti RL Kind AB Russell K Schoetzau A Heinzelmann-Schwarz VA. High-Grade Serous Peritoneal Cancer Follows a High Stromal Response Signature and Shows Worse Outcome Than Ovarian Cancer. Mol Oncol (2021) 91–103. doi: 10.1002/1878-0261.12811 33016563 PMC7782088

[5] Dai G Liang K Xiao Z Yang Q Yang S-G. A Meta-Analysis on the Diagnostic Value of Diffusion-Weighted Imaging on Ovarian Cancer. J Buon (2019) 2333–40. 31983103

[6] Deen SS Priest AN McLean MA Gill AB Brodie C Crawford R. Diffusion Kurtosis MRI as a Predictive Biomarker of Response to Neoadjuvant Chemotherapy in High Grade Serous Ovarian Cancer. Sci Rep (2019) 9(1):10742. doi: 10.1038/s41598-019-47195-4 31341212 PMC6656714

[7] Rizzo S De Piano F Buscarino V Pagan E Bagnardi V Zanagnolo V. Pre-Operative Evaluation of Epithelial Ovarian Cancer Patients: Role of Whole Body Diffusion Weighted Imaging MR and CT Scans in the Selection of Patients Suitable for Primary Debulking Surgery. A Single-Centre Study. Eur J Radiol (2020) 123:108786. doi: 10.1016/j.ejrad.2019.108786 31862634

[8] Kyriazi S Collins DJ Messiou C Pennert K Davidson RL Giles SL. Metastatic Ovarian and Primary Peritoneal Cancer: Assessing Chemotherapy Response With Diffusion-Weighted MR Imaging—Value of Histogram Analysis of Apparent Diffusion Coefficients. Radiology (2011) 261(1):182−92. doi: 10.1148/radiol.11110577 21828186

[9] Kumar V Gu Y Basu S Berglund A Eschrich SA Schabath MB. Radiomics: The Process and the Challenges. Magn Reson Imaging (2012) 30(9):1234−48. doi: 10.1016/j.mri.2012.06.010 22898692 PMC3563280

[10] Lambin P Rios-Velazquez E Leijenaar R Carvalho S van Stiphout RGPM Granton P. Radiomics: Extracting More Information From Medical Images Using Advanced Feature Analysis. Eur J Cancer (2012) 48(4):441−6. doi: 10.1016/j.ejca.2011.11.036 22257792 PMC4533986

[11] Rizzo S Botta F Raimondi S Origgi D Buscarino V Colarieti A. Radiomics of High-Grade Serous Ovarian Cancer: Association Between Quantitative CT Features, Residual Tumour and Disease Progression Within 12 Months. Eur Radiol (2018) 28(11):4849−59. doi: 10.1007/s00330-018-5389-z 29737390

[12] Nougaret S Tardieu M Vargas HA Reinhold C Vande Perre S Bonanno N. Ovarian Cancer: An Update on Imaging in the Era of Radiomics. Diagn Interventional Imaging (2019) 100(10):647−55. doi: 10.1016/j.diii.2018.11.007 30555018

[13] Song X Ren J-L Zhao D Wang L Ren H Niu J. Radiomics Derived From Dynamic Contrast-Enhanced MRI Pharmacokinetic Protocol Features: The Value of Precision Diagnosis Ovarian Neoplasms. Eur Radiol (2021) 31(1):368−78. doi: 10.1007/s00330-020-07112-0 32767049

[14] Vargas HA Veeraraghavan H Micco M Nougaret S Lakhman Y Meier AA. A Novel Representation of Inter-Site Tumour Heterogeneity From Pre-Treatment Computed Tomography Textures Classifies Ovarian Cancers by Clinical Outcome. Eur Radiol (2017) 27(9):3991−4001. doi: 10.1007/s00330-017-4779-y 28289945 PMC5545058

[15] Athavale R Thomakos N Godfrey K Kew F Cross P Lopes ADB. The Effect of Epithelial and Stromal Tumor Components on FIGO Stages III and IV Ovarian Carcinosarcomas Treated With Primary Surgery and Chemotherapy. Int J Gynecologic Cancer (2007) 17(5). doi: 10.1111/j.1525-1438.2007.00919.x 17466043

[16] Labiche A Heutte N Herlin P Chasle J Gauduchon P Elie N. Stromal Compartment as a Survival Prognostic Factor in Advanced Ovarian Carcinoma. Int J Gynecologic Cancer (2010) 20(1). doi: 10.1111/IGC.0b013e3181bda1cb 20130500

[17] Greenaway J Moorehead R Shaw P Petrik J. Epithelial–stromal Interaction Increases Cell Proliferation, Survival and Tumorigenicity in a Mouse Model of Human Epithelial Ovarian Cancer. Gynecologic Oncol (2008) 108(2):385−94. doi: 10.1016/j.ygyno.2007.10.035 18036641

[18] Lu H Arshad M Thornton A Avesani G Cunnea P Curry E. A Mathematical-Descriptor of Tumor-Mesoscopic-Structure From Computed-Tomography Images Annotates Prognostic- and Molecular-Phenotypes of Epithelial Ovarian Cancer. Nat Commun (2019) 10(1):764. doi: 10.1038/s41467-019-08718-9 30770825 PMC6377605

[19] Dashevsky BZ D’Alfonso T Sutton EJ Giambrone A Aronowitz E Morris EA. The Potential of High Resolution Magnetic Resonance Microscopy in the Pathologic Analysis of Resected Breast and Lymph Tissue. Sci Rep (2015) 5:17435. doi: 10.1038/srep17435 26639673 PMC4671009

[20] Fan X Haney CR Agrawal G Pelizzari CA Antic T Eggener SE. High-Resolution MRI of Excised Human Prostate Specimens Acquired With 9.4T in Detection and Identification of Cancers: Validation of a Technique. J Magn Reson Imaging (2011) 34(4):956−61. doi: 10.1002/jmri.22745 21928309

[21] Durand M Jain M Robinson B Aronowitz E Douahy YE Leung R. Magnetic Resonance Microscopy may Enable Distinction Between Normal Histomorphological Features and Prostate Cancer in the Resected Prostate Gland. BJU Int (2017) 119(3):414−23. doi: 10.1111/bju.13523 27154761

[22] Heidkamp J Hoogenboom M Kovacs IE Veltien A Maat A Sedelaar JPM. *Ex Vivo* MRI Evaluation of Prostate Cancer: Localization and Margin Status Prediction of Prostate Cancer in Fresh Radical Prostatectomy Specimens. J Magn Reson Imaging (2018) 47(2):439−48. doi: 10.1002/jmri.25785 28580659

[23] Martínez-Bisbal MC Martínez-Granados B Rovira V Celda B Esteve V. Magnetic Resonance Spectroscopy and Imaging on Fresh Human Brain Tumor Biopsies at Microscopic Resolution. Anal Bioanal Chem (2015) 407(22):6771−80. doi: 10.1007/s00216-015-8847-3 26123440

[24] Coillot C Sidiboulenouar R Nativel E Zanca M Alibert E Cardoso M. Signal Modeling of an MRI Ribbon Solenoid Coil Dedicated to Spinal Cord Injury Investigations. J Sens Sens Syst (2016) 5:137−45. doi: 10.5194/jsss-5-137-2016

[25] Mayerhoefer ME Szomolanyi P Jirak D Berg A Materka A Dirisamer A. Effects of Magnetic Resonance Image Interpolation on the Results of Texture-Based Pattern Classification: A Phantom Study. Invest Radiol (2009) 44(7):405−11. doi: 10.1097/RLI.0b013e3181a50a66 19465863

[26] Zwanenburg A Leger S Vallières M Löck S. Initiative for the IBS. Image Biomarker Standardisation Initiative. arXiv (2016) 161207003. doi: 10.1148/radiol.2020191145

[27] Fedorov A Beichel R Kalpathy-Cramer J Finet J Fillion-Robin J-C Pujol S. 3d Slicer as an Image Computing Platform for the Quantitative Imaging Network. Magn Reson Imaging (2012) 30(9):1323−41. doi: 10.1016/j.mri.2012.05.001 22770690 PMC3466397

[28] Haralick RM Shanmugam K Dinstein I. Textural Features for Image Classification. IEEE Trans Syst Man Cybern (1973) SMC-3(6):610−21. doi: 10.1109/TSMC.1973.4309314

[29] Bankhead P Loughrey MB Fernández JA Dombrowski Y McArt DG Dunne PD. QuPath: Open Source Software for Digital Pathology Image Analysis. Sci Rep (2017) 7(1):16878. doi: 10.1038/s41598-017-17204-5 29203879 PMC5715110

[30] Gibson E Crukley C Gaed M Gómez JA Moussa M Chin JL. Registration of Prostate Histology Images to *Ex Vivo* MR Images *via* Strand-Shaped Fiducials. J Magn Reson Imaging (2012) 36(6):1402−12. doi: 10.1002/jmri.23767 22851455

[31] Lopater J Colin P Beuvon F Sibony M Dalimier E Cornud F. Real-Time Cancer Diagnosis During Prostate Biopsy: *Ex Vivo* Evaluation of Full-Field Optical Coherence Tomography (FFOCT) Imaging on Biopsy Cores. World J Urol (2016) 34(2):237−43. doi: 10.1007/s00345-015-1620-6 26100944

