# Supplementary figures and images for: Assessing Histology Structures by Ex Vivo MR Microscopy and Exploring the Link Between MRM-Derived Radiomic Features and Histopathology in Ovarian Cancer
Source: Front Oncol. 2022 Jan 19;11:771848. doi: 10.3389/fonc.2021.771848 (PMC8807492; doi:10.3389/fonc.2021.771848)

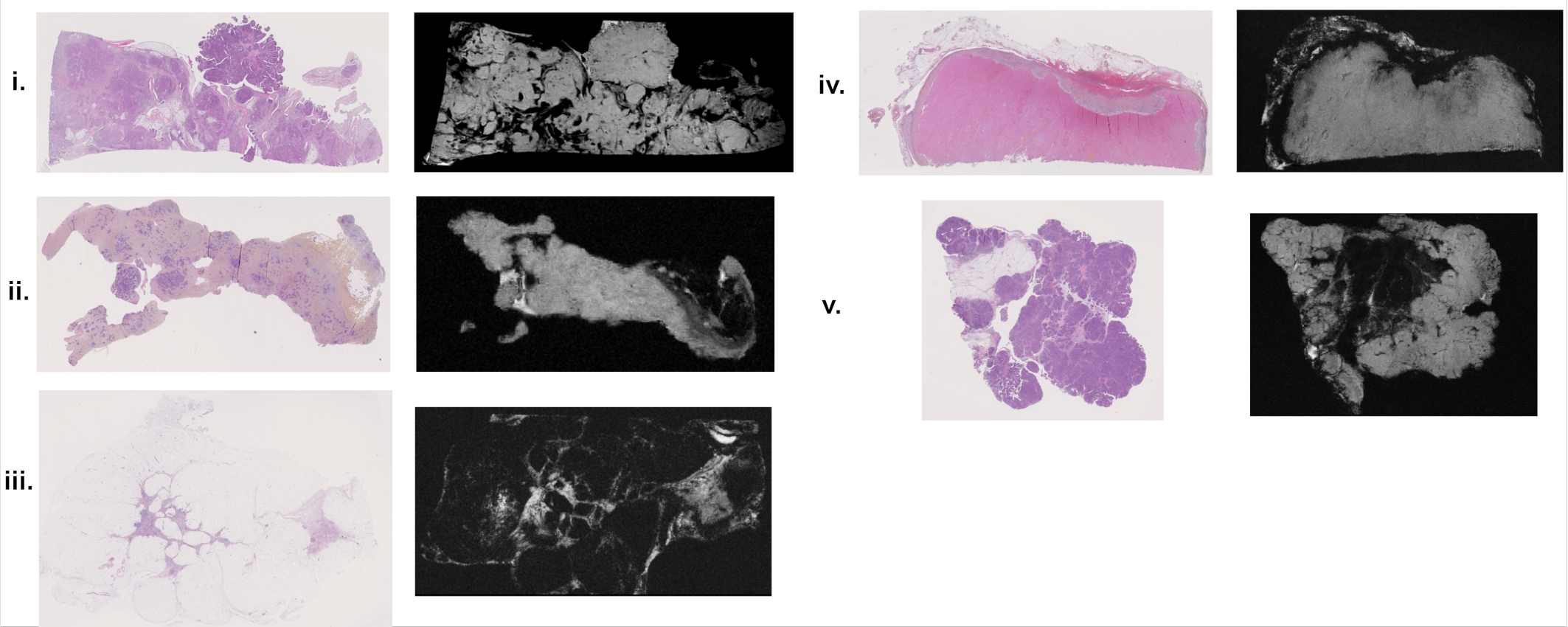

Supplement: Supplementary Figure 1 — H&E stained histological images (left) with corresponding high-resolution MR images (right), for 5 of the 9 resected peritoneal implants (i. to v.). [file Image_1.tiff]

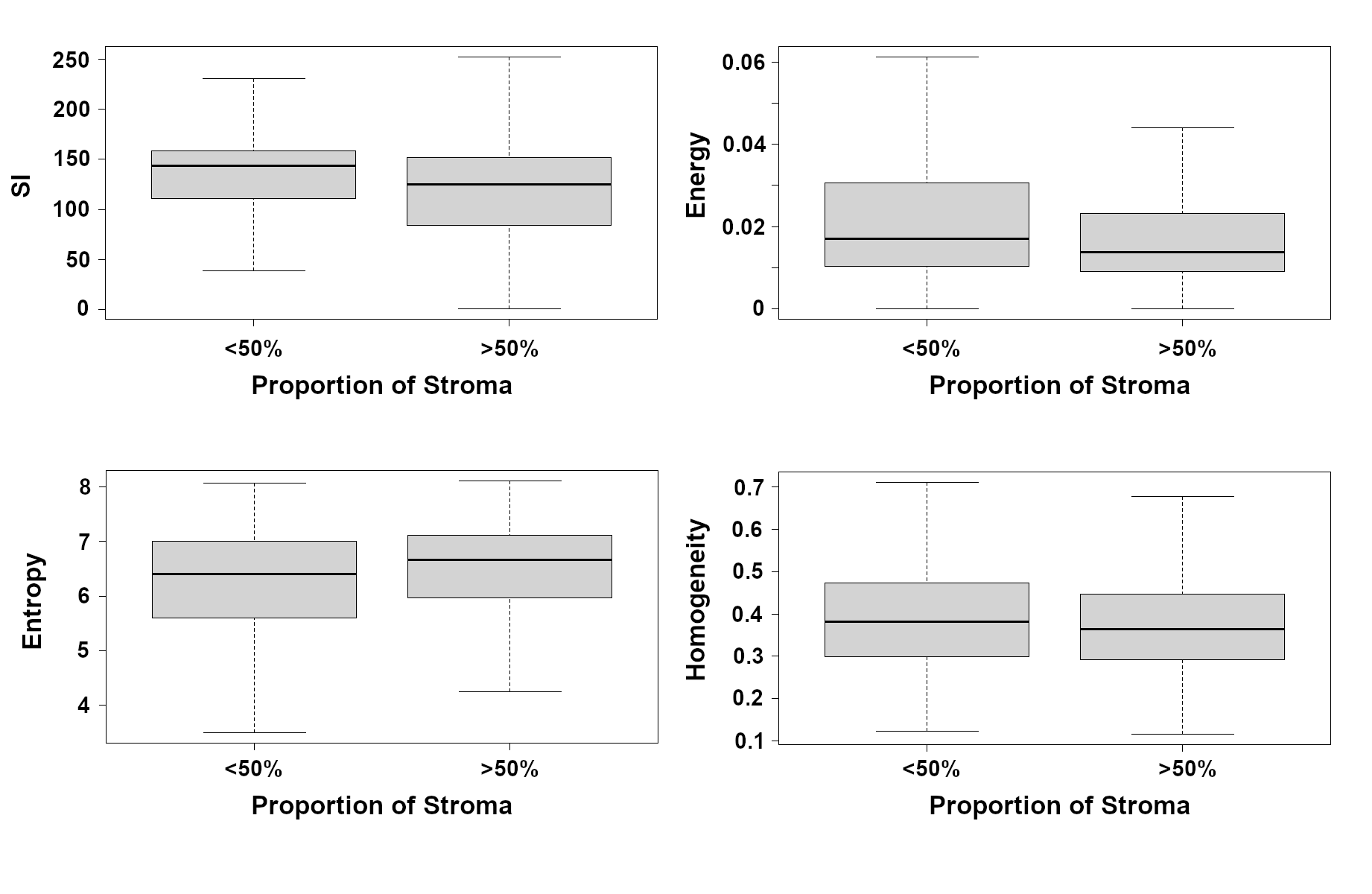

Supplement: Supplementary Figure 2 — Box plots showing feature values between pixels labeled as stromal proportion greater than 50% and as less than 50%, measured from the four available HGSOC peritoneal implants (total of 196073 classified pixels). Wilcoxon test, p-values <0.0001, for the 4 presented features. [file Image_2.tiff]
